# Dengue virus serotypic replacement of NS3 protease or helicase domain causes chimeric viral attenuation but can be recovered by a compensated mutation at helicase domain or NS2B, respectively

**DOI:** 10.1128/jvi.00854-23

**Published:** 2023-08-09

**Authors:** Tadahisa Teramoto

**Affiliations:** 1 Department of Microbiology and Immunology, Georgetown University, Washington, DC, USA; Cornell University Baker Institute for Animal Health, Ithaca, New York, USA

**Keywords:** dengue virus, NS2B, NS3, protease, helicase, chimeric virus, serotypic replacement, mosquito

## Abstract

Mosquito-borne dengue viruses (DENVs) have evolved to four serotypes with 69%–78% amino acid identities, resulting in incomplete immunity, where one serotype’s infection does not cross-protect against secondary infections by other serotypes. Despite the amino acid differences, structural and nonstructural (NS) proteins among serotypes play similar functions. NS3 is an enzyme complex: NS3 has N-terminal protease (PRO) and C-terminal helicase (HEL) activities in addition to 5’ RNA triphosphatase (5’RTP), which is involved in the RNA capping process. In this study, the effects of NS3 replacements among serotypes were tested. The replacement of NS3 full-length (FULL), PRO or HEL region suppressed viral replication in BHK-21 mammalian cells, while the single compensatory mutation improved the viral replications; P364S mutation in HEL revived PRO (DENV3)-replaced DENV1, while S68T alteration in NS2B recovered HEL (DENV1)-replaced DENV2. The results suggest that the interactions between PRO and HEL as well as HEL and NS2B are required for replication competence. Lower-frequency mutations also appeared at various locations in viral proteins, although after infecting C6/36 mosquito cells, the mutations’ frequencies changed, and/or new mutations appeared. In contrast, the inter-domain region (INT, 12 amino acids)-replaced chimera quickly replicated without mutation in BHK-21 cells, although extended cell culture accumulated various mutations. These results suggest that NS3 variously interacts with DENV proteins, in which the chimeric NS3 domain replacements induced amino acid mutations, irrespective of replication efficiency. However, the viral sequences are further adjusted for replication efficiency, to fit in both mammalian cells and mosquito cells.

**IMPORTANCE:**

Enzyme activities for replicating DENV 5’ cap positive (+) sense RNA have been shown to reside in NS3 and NS5. However, it remains unknown how these enzymes coordinately synthesize negative (-) sense RNA, from which abundant 5’ cap (+) sense RNA is produced. We previously revealed that NS5 dimerization and NS5 methyltransferase(MT)–NS3HEL interaction are important for DENV replication. Here, we found that replication incompetence due to NS3PRO or HEL replacement was compensated by a mutation at HEL or NS2B, respectively, suggesting that the interactions among NS2B, NS3PRO, and HEL are critical for DENV replication.

## INTRODUCTION

Arthropod-borne viruses (Arboviruses), including flaviviruses and alphaviruses, have evolved by serially infecting vertebrates and arthropods (1). Vertebrate hosts are mammals and birds, while the arthropod vectors are mostly mosquitoes, or for some flaviviruses, ticks. The human–mosquito transmission cycle is established for dengue virus four serotypes (DENV1–4) due to cross-contacts between human and mosquitoes (2). The primary mosquito vectors are *Aedes aegypti* and *Aedes albopictus*, both of which are expanding their habitats from their original tropical and subtropical areas with the help of increased global warming (3).

Flavivirus genomes are 5’ cap (+) sense RNAs, coding single polyproteins consisting of N-terminal three structural and C-terminal seven NS proteins. The boundaries among structural proteins and NS1, in addition to N-terminal NS4B, are cleaved by cellular signalase at the endoplasmic reticulum (ER) membrane (4 - 7), while NS2A to NS5 boundaries are cleaved by the viral protease (NS2B-activated NS3PRO) (8, 9). By the coordinated functions of the NS3 and NS5 enzyme complex, the viral RNA is reproduced at the ER transformed by the viral membrane-integral proteins (NS2A, NS2B, NS4A, and NS4B) (10). Transmembrane domains (TMDs) in these NS proteins provide support for replicating viral RNA and packaging the synthesized RNA into the particle. Both processes are impaired by single amino acid alterations in NS2A (11). NS2B has a central hydrophilic region activating NS3PRO (12) and flanking hydrophobic regions containing TMDs that are also involved in the viral RNA synthesis and packaging (13). NS4A-NS4B boundary releases 2K peptide by cleavage with NS2B-NS3PRO and cellular signalase (7). The 2K release initiates the rearrangement of ER membrane and distributes NS4A to the Golgi apparatus, which leads to an ER-to-Golgi trafficking, a necessary step for packaging the viral RNA into the viral particle (14, 15). It has been reported that amino acid mutations, which abolish an interaction between NS4A and NS4B or between NS3 and NS4B, disable viral RNA replications (16, 17).

NS3 and NS5 are enzyme complex proteins that cover all the processes for reproducing the viral genome. NS3 consists of N-terminal PRO and C-terminal HEL in addition to 5’ RNA triphosphatase (5’RTP), which initiates the first step for 5’ capping of the newly synthesized (+) sense RNA by removing a phosphate from 5’ terminal “pppA” nucleotide to create “ppA” (18). Guanylyltransferase and MT, both of which reside at N-terminal NS5, continue the capping process by sequentially generating GpppA and m^7^GpppAm (guanine N-7 methylation preceding ribose 2′-*O* methylation) at the 5’ terminus (19, 20). NS5 C-terminal polymerase(POL) uses the input (+) sense viral RNA for synthesizing (-) sense RNA, which in turn after being separated from the (+) sense RNA by NS3HEL, is utilized as a template for repeatedly producing (+) sense RNA. The mechanism behind the excess synthesis of viral (+) sense RNA was partly shown in the plant Tombusvirus, in which the replication enhancer sequences in (-) sense RNA selectively drove (+) sense RNA synthesis (21). In flaviviruses, the mechanism remains unclarified whether the similar regulatory enhancer exists in (-) sense RNA or other elements exist, such as RNA replication silencer in (+) sense RNA being present. Cellular and/or viral proteins including NS5POL supposedly respond to these sequences on viral (+) or (-) sense RNA.

During our previous studies of NS5MT or POL domain replacement between DENV2 and DENV4, we found that the substitution of either MT or POL domain delayed the viral reproductions despite the preserved MT and POL enzyme activities in the chimeric NS5 (22, 23). However, after viral replication appeared at ~14 d post electroporation (p.e.), MT-replaced chimeric viruses gradually expanded and had acquired a single compensated mutation at K74I in MT or D290N in NS3HEL (22). POL-replaced chimeric viruses also delayed the replication but later expanded with compensated mutations at D51N and/or K76I/R in MT, in addition to lower-frequency (30 ~ 50%) mutations in the areas of the NS5 dimer interface (23). The data indicated that the delay in NS5-chimeric viral replication was due to the loss of interactions between NS3HEL and NS5MT as well as between MT and POL in NS5, which forms dimerization. In this study, we similarly analyzed the impact of NS3 domain replacement (FULL, PRO, HEL, or INT) in viral replication and the viral genome sequence in order to reveal the NS3 function in replication processes.

## RESULTS

### The replacement of NS3FULL, PRO, HEL, or INT caused viral attenuation

**Fig 1:**
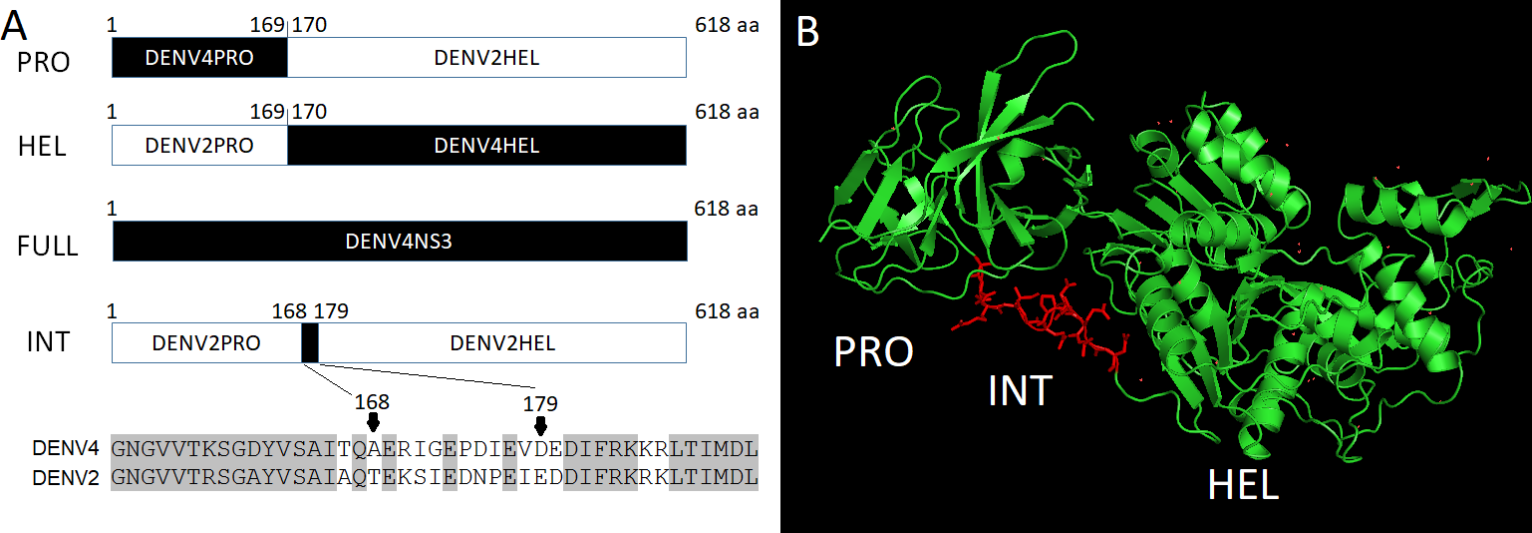
NS3 replacements are designed by targeting enzyme domains. (**A**) DENV2 NS3 PRO, HEL, FULL, and INT regions were substituted by the corresponding in DENV4. Amino acid numbers (1–618 aa) in NS3 are described. The whole NS3 618~619 amino acid alignment among four serotypes and four DENV2 strains are compared in Fig. S1 and S2, respectively. Alignment of the amino acid sequences of INT are depicted on the bottom. Identical amino acid residues are shaded in gray. (**B**) DENV4 NS3 crystal structure (PDB:2VBC). PRO (green color), INT (red color), and HEL (green color) regions are shown.

**Fig 2:**
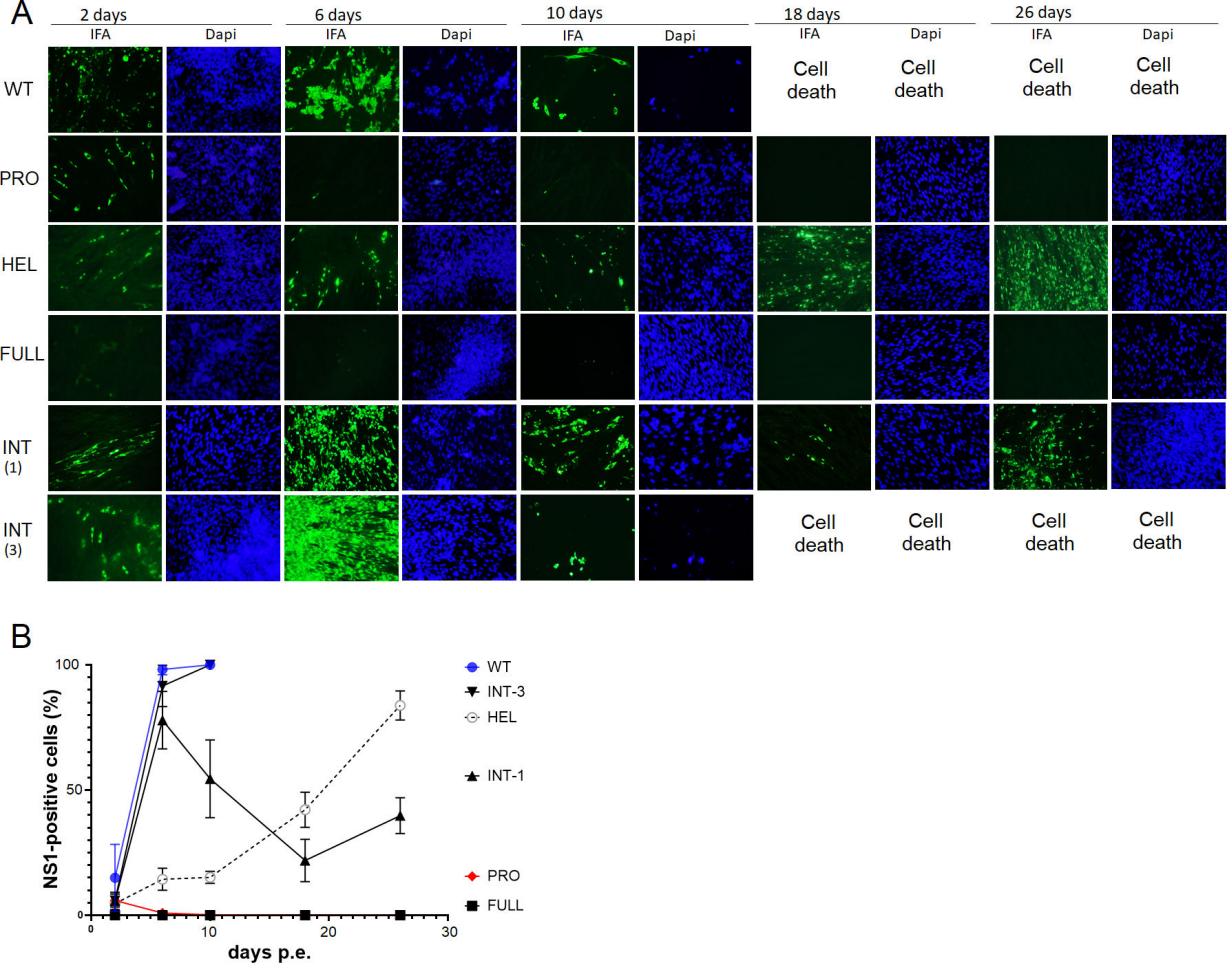
Viral replication was monitored by immunofluorescent staining against DENV NS1. (**A**) FITC (green fluorescent)-labeled secondary antibody was used to detect the primary anti-NS1 antibody. IFA: immunofluorescent staining against NS1. Dapi: Nuclear staining by DAPI solution (blue fluorescence). Both images were compared among WT and chimeric electroporated cells. (**B**) The rate of NS1-positive cells per all cells in the microscopic fields were calculated using ImageJ software and compared among samples and days p.e. The numbers represent mean ± SD (*N* = 6).

The sequence identities of DENV polyproteins among four serotypes are lowest between DENV2 and DENV4 (Table 1). NS3FULL, PRO, HEL, or INT in DENV2 full-length cDNA was replaced by each corresponding sequence from DENV4 (Fig. 1), creating chimeric proteins that consist of lower amino acids. Each chimeric cDNA was constructed through the yeast recombination method and was used for synthesizing each infectious RNA by *in vitro* transcription reaction with a cap analog. The synthesized RNA was electroporated into BHK-21 mammalian cells and monitored for replication efficiency by immunofluorescent (IF) staining against viral NS1. The wild-type (WT) DENV2 RNA replicated quickly at 2 days p.e. and induced cell death, accompanied with fragmented nuclei (cell death) shown by Dapi staining at 6 days p.e. (Fig. 2). The WT DENV2 RNA made it difficult to continue culturing in BHK-21 cells beyond 10 days p.e. In contrast, PRO, HEL, or INT-replaced chimeric RNA induced fewer viral positive cells compared with WT DENV2 and delayed its spread to all cells (Fig. 2). Among these chimera, PRO-replaced virus did not expand through the extended cell culture beyond 10 days p.e. and disappeared, while INT chimera quickly expanded during the 6–10 days p.e. (Fig. 2). Among triplicated repetitions, the different features of viral spreads were observed in INT-chimeric RNAs. In one experiment, it was difficult to continue cell culture beyond 10 days due to more severe cell death and aggressively reduced cell numbers (INT (3), Fig. 2), while the other two experiments enabled extended cultures due to less cytotoxicity and stalled expansion of the viral progenies at later time points (INT (1), Fig. 2). HEL-chimeric viruses showed slower expansion than INT chimera in all triplicated experiments. HEL chimeras gradually expanded to BHK-21 cells but lacked cytotoxicity to the cells (Fig. 2). In contrast, NS3FULL-replaced chimera did not show NS1 positivity in the cytoplasm by IF staining through all time points (Fig. 2).

**TABLE 1:** DENV protein homologies among serotypes

Serotypic comparison	Whole polyprotein identity	NS3 identity	NS5 identity
1 vs 2	72%	79%	79%
1 vs 3	78%	85%	82%
1 vs 4	69%	76%	76%
2 vs 3	72%	80%	78%
2 vs 4	69%	77%	74%
3 vs 4	70%	80%	77%

### NS3FULL or PRO viral RNA copy numbers were significantly lower than those in WT or INT, while HEL viral copy numbers slowly increased

**Fig 3:**
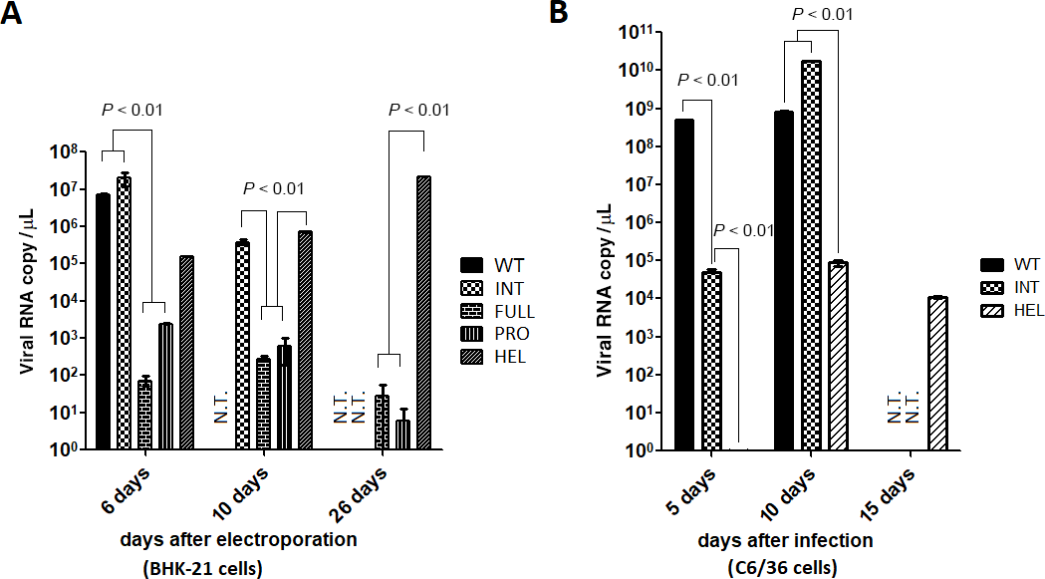
Viral RNA copy numbers in the cell culture supernatants were measured by RT-qPCR. Supernatants were collected at the indicated time points and used for extracting total RNA. After the reverse transcriptase reaction to make cDNA, quantitated PCR was performed with standard cDNA samples to determine the copy numbers. (**A**) Viral RNA copies from electroporated BHK-21 cells. (**B**) Viral RNA copies from infected C6/36 cells. N.T., not tested due to the lack of samples by cytotoxicity. The copy numbers represent mean values ± SD (*n* = 2). *P* < 0.01 was confirmed by one-way ANOVA with Bonferroni post-test.

INT chimera showed the highest copy numbers among NS3 chimera at 6 days p.e. (Fig. 3A). HEL chimera’s copy numbers were the next most abundant and reached its highest copy numbers at 26 days p.e., comparable to those of WT at 6 days p.e. (Fig. 3A). In contrast, PRO and FULL chimeras produced significantly lower copy numbers and decreased at 26 days p.e. (Fig. 3A).

### Infection of HEL-chimeric viruses to C6/36 mosquito cells showed lower replication than those with infections by WT or INT, did not induce C6/36-syncytial change**,** and lacked the plaque-forming cytotoxicity to LLC/MK2 mammalian cells

**Fig 4:**
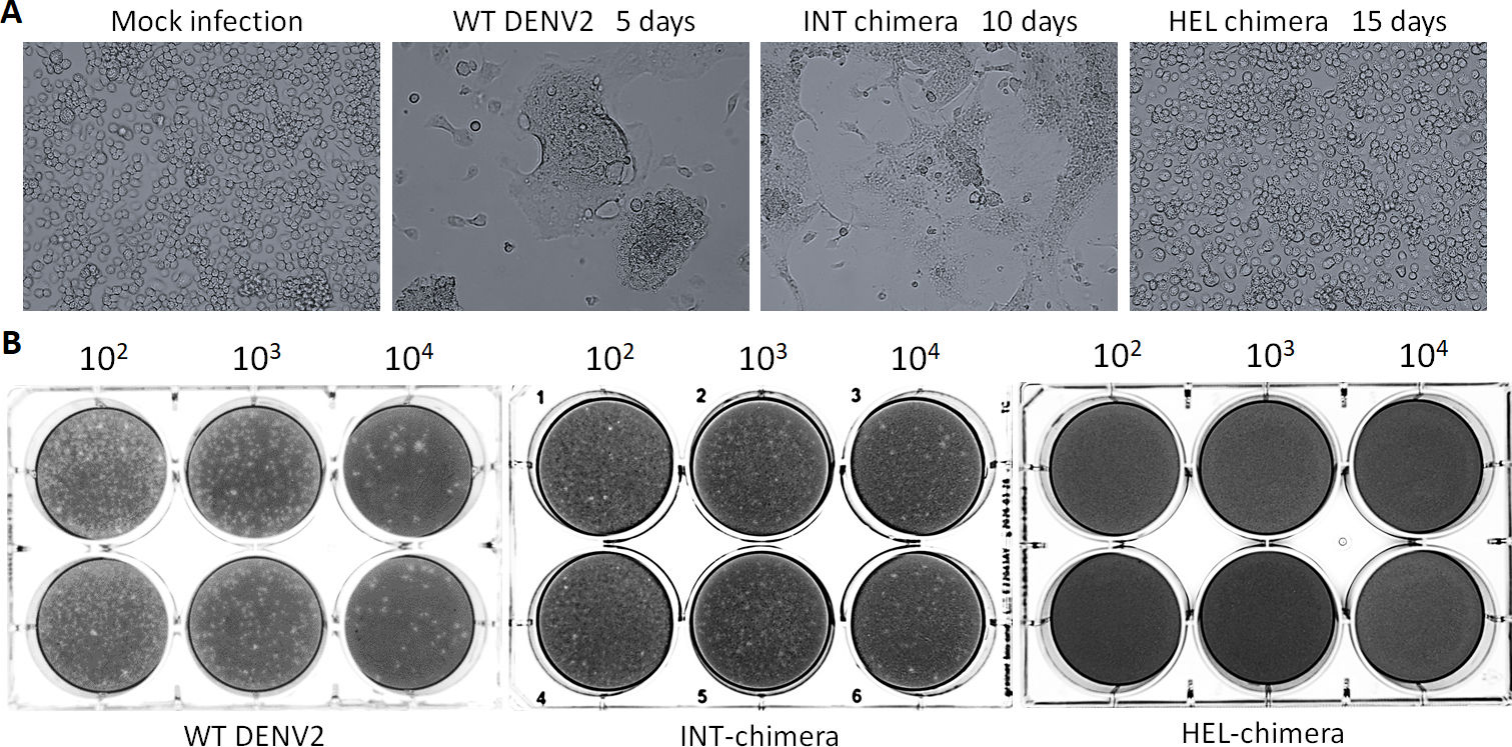
The viruses recovered from HEL replacement did not induce the syncytial formation of C6/36 cells, nor cytotoxic plaque formation in LLC/MK2 cells. (**A**) C6/36 cells were infected by viruses collected from supernatants of WT, INT, or HEL RNA-electroporated BHK-21 cells. Light microscopic views were taken on the indicated days. (**B**) Plaque assays were done on duplicated wells (top and bottom) using LLC/MK2 cells infected by viruses collected after infecting C6/36 (at the days shown in Fig. 4A). Dilution factors are shown at the top.

The equal viral copy numbers among WT, INT, and HEL chimera viruses (collected from BHK-21 cell culture supernatants at 6, 6, and 26 days p.e., respectively) were used for infecting C6/36 mosquito (*Aedes albopictus*) cells. INT-chimeric viruses replicated at lower copy numbers compared with WT at 5 days post infection (p.i.) but reached the WT DENV2 copy numbers at 10 days p.i. (Fig. 3B). In contrast, HEL-chimeric viruses replicated with the lowest copy numbers through all time points (Fig. 3B). DENV2 is known to induce syncytium-forming morphological change of C6/36 cells (24). WT- or INT-replaced DENV2 induced syncytial change of C6/36 cells at ~5 days or ~10 days p.i., respectively, while HEL-replaced DENV2 did not show a clear morphological change (Fig. 4A). WT- or INT-chimeric viruses (collected at 5 and 10 days p.i., respectively) induced cell death in LLC/MK2 mammalian cells in plaque assays, while HEL-replaced chimeric viruses did not (Fig. 4B). These results indicate that HEL-replaced DENV2 possesses significantly less replication competence and lowered cytotoxicity, compared with WT or INT chimera.

### Reporter-DENV2 replicon RNA assay showed that the FULL-, PRO-, or HEL-chimeric replicon did not efficiently replicate, compared with the WT- or INT-chimeric one

**Fig 5:**
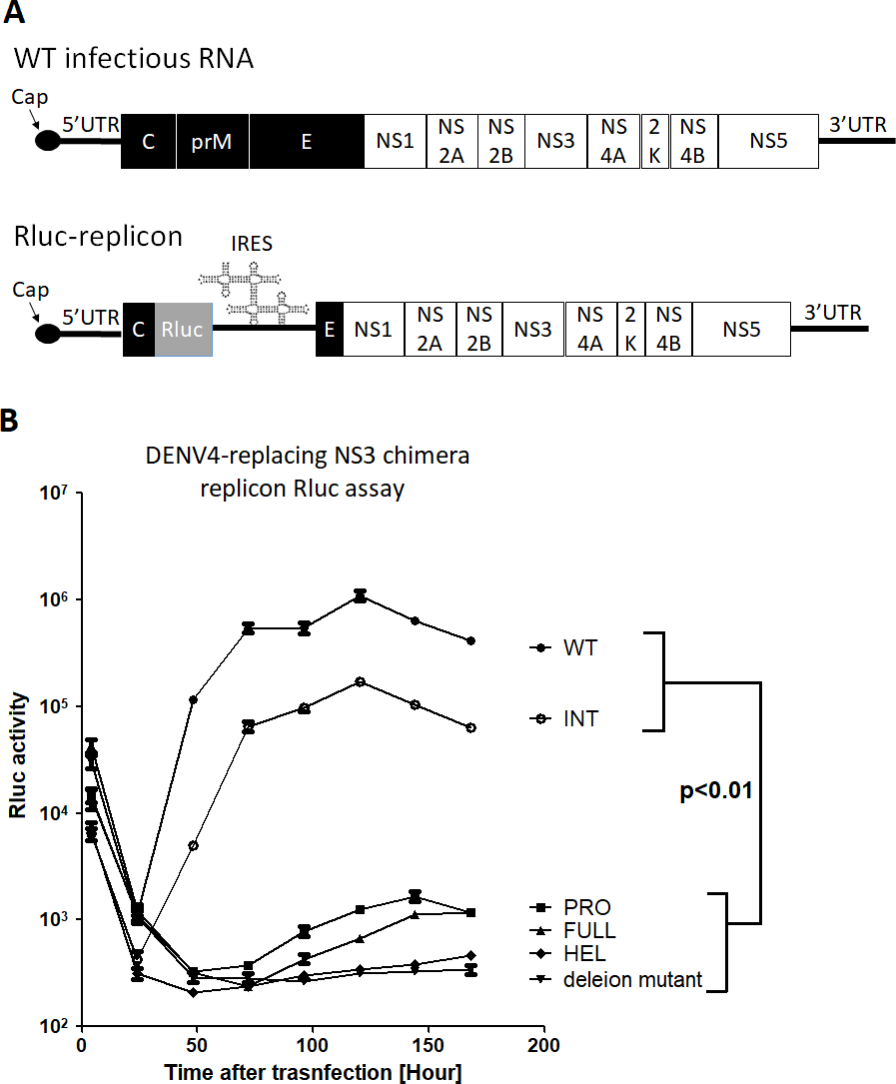
DENV2-Rluc reporter replicons among WT and chimeric RNAs are compared for the replication efficiency. (**A**) Designs and constructions of infectious DENV RNA (top) and Rluc-reporter replicon RNA (bottom). Replicon RNAs lack the viral structural regions but instead contain Rluc reporter gene, followed by EMCV IRES sequence. (**B**) Rluc assays were performed (Promega) with cell lysates extracted at 4, 24, 48, 72, 96, 120, 144, and 168 hours after transfection (electroporation). Rluc assays showed that WT- or INT-replicon replicated significantly faster than other NS3-chimeric replicons. *P* < 0.01 was confirmed by one-way ANOVA with Bonferroni post-test.

Renilla luciferase (Rluc)-contained DENV2 replicon cDNA was constructed to solely evaluate the RNA replication efficiency and to distinguish it from other processes, including the particle formation by structural proteins and the packaging of the synthesized RNA into the particle. The structural polyprotein regions in those infectious cDNAs were replaced by Rluc. Then, IRES sequence was inserted prior to coding NS polyproteins (Fig. 5A). The *in vitro* transcribed 5’ cap RNAs were electroporated into BHK-21 cells and Rluc activities were measured periodically. At 4 hours after electroporation, all replicon RNAs could induce the initial Rluc activity peaks derived from the translation of the input RNAs (Fig. 5B). At 24 hours, this peak decreased due to the decays of the input RNA. After that, Rluc activities of WT DENV2 replicon increased as a result of the increased newly synthesized replicon RNAs. In contrast, INT-chimeric replicon showed less Rluc activities, reflecting the less synthesized replicon RNAs. Rluc activities of FULL-, PRO-, or HEL-chimeric replicon were notably even lower. This replicon data indicated that all NS3-chimeric replacements except INT significantly lowered the synthesis of the replicon RNA.

### The PRO chimera, replaced by DENV1 or DENV3, as well as the HEL chimera by DENV1 were also unable to expand after the initial replication, while HEL chimera by DENV3 could expand slowly

**Fig 6:**
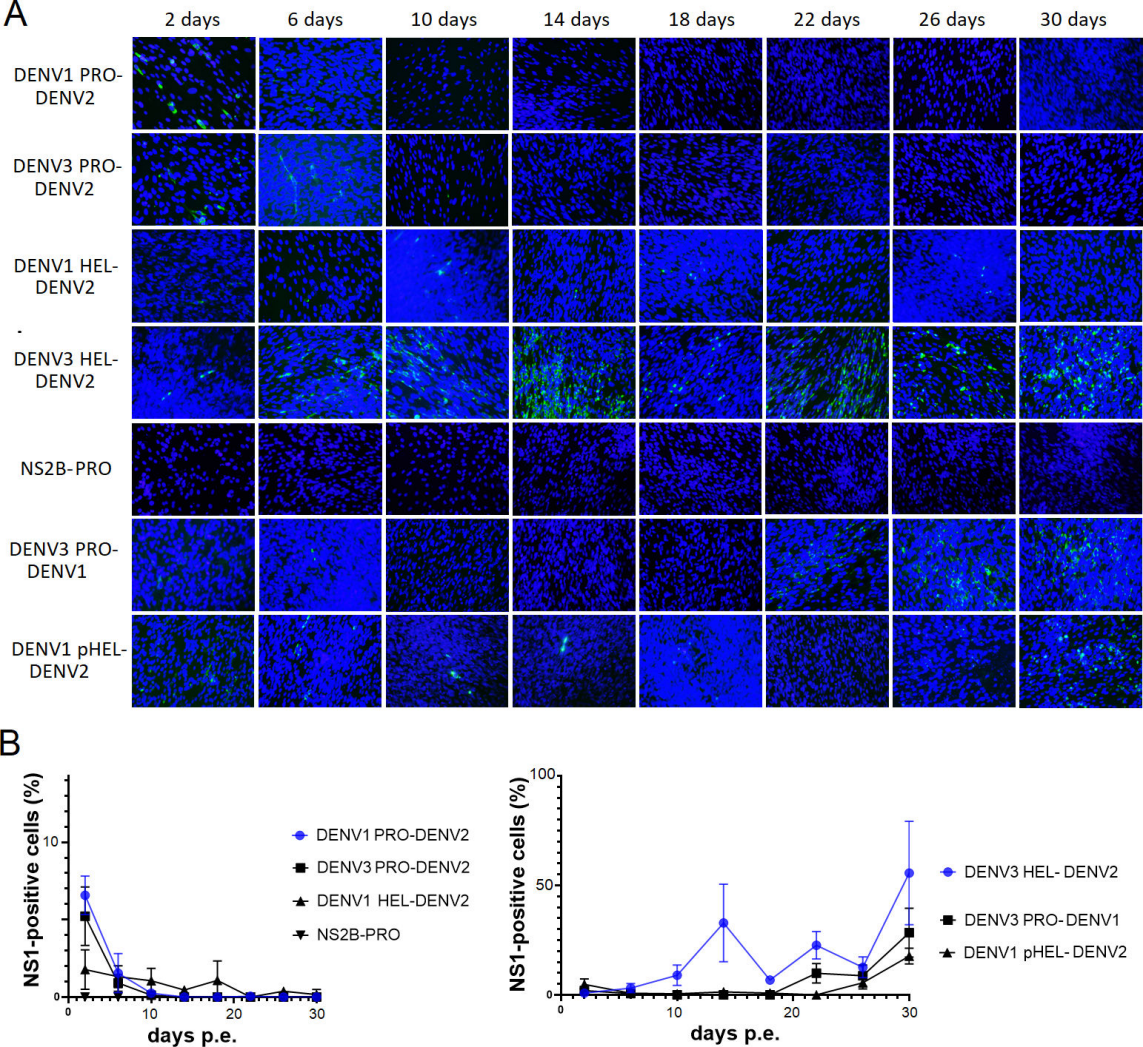
Further replacements of NS3 domains in DENV2 were created with DENV1 or DENV3 corresponding (DENV1 PRO-DENV2, DENV3 PRO-DENV2, DENV1 HEL-DENV2, and DENV3 HEL-DENV2). Additional replacement of NS2B with DENV4 corresponding was also tested (NS2B-PRO). DENV1 backbone was used for PRO replacement with DENV3 corresponding to make the highest homology (DENV3 PRO-DENV1). N-terminal partial replacement of HEL (pHEL) was created in DENV1 HEL replacement (DENV1 pHEL-DENV2). These chimeric viral replications were monitored after the RNA electroporations into BHK-21 cells. (**A**) IF staining (green fluorescent) against DENV NS1 and Dapi nuclei staining (blue fluorescence) were merged to clarify the cellular localization of NS1. (**B**) The rate of NS1-stained cells in the samples were measured at each time point of the microscopic fields and were compared.

In order to confirm whether the chimeric viral expansion depends on the region of the replacement, the infectious DENV2 cDNA was alternately replaced in its PRO or HEL region by the corresponding one from DENV1 or DENV3. PRO-chimeric RNA from DENV1 or DENV3 showed the disappearance of the viral protein NS1 after 6 days p.e. of the initial replication (Fig. 6). HEL-chimeric RNA from DENV1 stalled the viral expansion and disappeared at later time points (> 26 days) (Fig. 6), while HEL chimera by DENV3 could gradually expand, resembling HEL replacement by DENV4 (Fig. 2 and 6). These results suggest that the PRO region is more serotypic sequence-specific than the HEL sequence for its role in the replication process.

**Fig 7:**
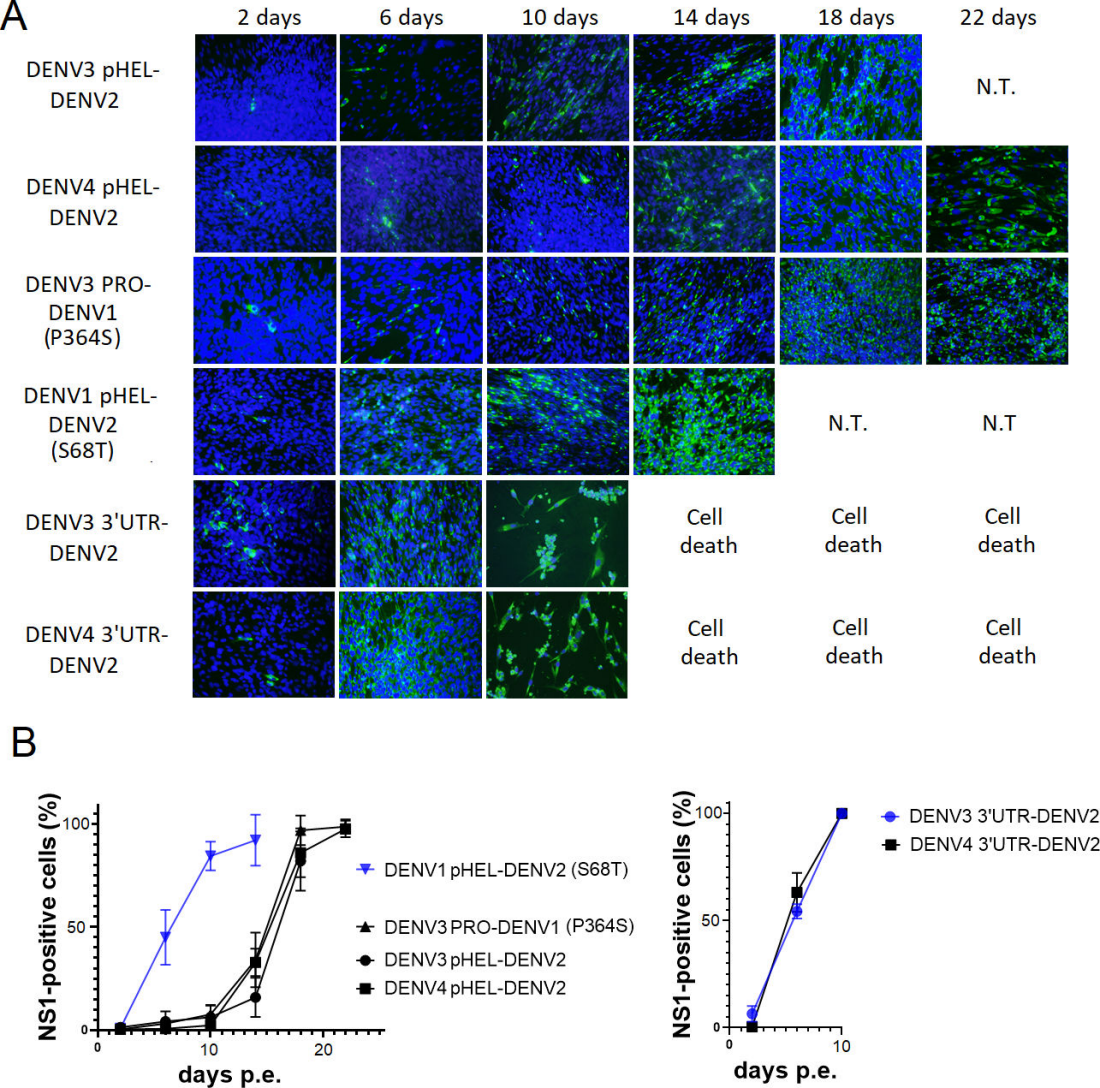
N-terminal partial replacement of HEL (pHEL) was created for DENV3 or DENV4 HEL replacement in DENV2 backbone RNAs (DENV3 pHEL-DENV2 and DENV4 pHEL-DENV2). Compensatory mutation-contained chimeric RNAs were made (DENV3 PRO-DENV1-P364S and DENV1 pHEL-DENV2-S68T). 3’UTR replacements in DENV2 backbone RNA with DENV3 or DENV4 corresponding were created (DENV3 3’UTR-DENV2 and DENV4 3‘UTR-DENV2). Chimeric viral replications were monitored after the RNA electroporations into BHK-21 cells. (**A**) Green fluorescent staining against DENV NS1 and nuclei blue fluorescent staining by DAPI solution were merged. N.T., not tested. (**B**) The rate of NS1-stained cells in the microscopic views were measured with ImageJ software and were compared.

### Combined replacement of NS2B and PRO did not revive replication competence

Since NS2B-NS3PRO functions as a cofactor-protease complex, it was considered that the same serotypic NS2B-NS3PRO sequence may be required for efficient viral RNA replication. The same serotypic replacements by DENV4 sequences were applied to create NS2B-NS3PRO-replaced infectious DENV2 cDNA. The *in vitro* transcribed 5’ cap chimeric RNA was electroporated into BHK-21 cells. The viral replication was not observed through all time points (Fig. 6).

### The PRO replacement using the corresponding from DENV3 for DENV1 infectious RNA gained replication efficiency at a later time point

In order to improve the viral replication of PRO-replaced RNA, PRO-replacement was designed between DENV1 and DENV3, both of which possess the highest NS3 amino acid identity (85%) among DENV serotypes (Table 1). DENV3 PRO-replacing DENV1 full-length cDNA was constructed. The transcribed 5’ cap chimeric RNA initially replicated in BHK-21 cells during 2–6 days p.e., although after that it became undetectable in IFA (Fig. 6). However, the virus replication reappeared at a later time point (~22 days p.e.) and gradually expanded its spread through cells (Fig. 6). This result indicated that the virus started to grow only after it acquired higher replication competency at a later time point.

### Reduced replacement region of HEL from DENV1 revived the chimeric DENV2 expansion at a later time point

It was also considered that a smaller replacement region of NS3 may improve viral progeny replication. The infectious DENV2 cDNA, in which partly N-terminal HEL (pHEL, 170–514 a.a) (Fig. 1) was replaced by the corresponding from DENV1, was used for *in vitro* transcription reaction. This 5’ capped pHEL-chimeric RNA showed stalled viral expansion and few NS-1 positive cells until 26 days p.e., similar to the complete HEL (DENV1) replacement, although after that the viral progenies gradually expanded (Fig. 6). This result indicated that the same serotypic C-terminal HEL to the backbone DENV serotype did not improve the stalled replication from the beginning but helped the expansion at a later time point. Partial HEL replacements in DENV2 with the corresponding from DENV3 (170–510 a.a.) or DENV4 (170–573 a.a.) showed the similar contribution to specially accelerate the expansion at later time points, compared to the fully HEL-replaced chimeric RNAs (Fig. 2, 6, and 7).

### HEL-chimeric viruses acquired various NS protein mutations with a wide range of frequencies

After viral expansions were confirmed by IFA, virus particles were collected from supernatants in cell cultures. The extracted viral RNAs were analyzed by next-generation RNA sequencing (RNAseq). As a control, WT DENV2 did not show an apparent (>5%) amino acid alteration in the electroporated BHK-21 cells as well as in the secondarily infected C6/36 cells (Table 2). In contrast, HEL replacement by DENV4 induced various amino acid mutations on structural ~NS proteins, including moderate-frequency mutations on NS2A and NS5MT (51% and 50%, respectively), and lower-frequency ones on NS2B (10%–16%) and NS3HEL (5%–15%) (Table 3). However, most of these amino acid alterations were not observed in the other two independently repeated experiments. Instead, alternative amino acid mutations appeared at NS5MT and NS5POL with various frequencies (7%–34%), while only one common but lower-frequency (5%–6%) amino acid mutation was observed (at E514K in HEL) (Table 3). The lack of high-frequency (>90%) amino acid alteration in all triplicated experiments indicates that the HEL replacement does not seriously affect replication and no compensatory mutation is required. In addition, infection of the HEL-chimeric DENV2 progeny to C6/36 mosquito cells altered the primary sequence variations; three mutations individually in E, NS5MT, and 3’ UTR disappeared, while new mutations in E, NS2B, and NS5POL appeared (Table 3).

**TABLE 2:** Nucleotide (amino acid) alteration in WT DENV2^*b*^

Host cell	BHK-21	C6/36
RNA	WT DENV2	WT DENV2 ^*a*^
Virus collection	10 days p.e.	11 days p.i.
5′-UTR	ND	ND
C	ND	ND
prM	ND	ND
E	ND	ND
NS1	ND	ND
NS2A	ND	ND
NS2B	4452 (S107 silent 11.6%)	ND
NS3PRO	ND	ND
NS3HEL	ND	ND
NS4A	ND	ND
NS4B	ND	ND
NS5MT	ND	ND
NS5POL	ND	ND
3′-UTR	ND	ND

^*a*^ The supernatant of the WT DENV2 RNA-electroporated BHK-21 cells was used.

^*b*^ The cutoff for inclusion is >5% abundance. The numbers refer to the nucleotide localization (1 to 10723) in DENV2 RNA, followed by the corresponding original amino acid, location, mutated amino acid, and frequency of occurrence shown within parentheses. ND, not detected.

**TABLE 3:** Nucleotide (amino acid) alteration in DENV4 HEL-chimeric DENV2^*b*^

Host cell	BHK-21	BHK-21	BHK-21	C6/36
RNA	HEL(DENV4)-DENV2 (1)	HEL(DENV4)-DENV2 (2)	HEL(DENV4)-DENV2 (3)	HEL(DENV4)-DENV2 (3)^*a*^
Virus collection	52 days p.e.	34 days p.e.	38 days p.e.	15 days p.i.
5′-UTR	ND	ND	ND	ND
C	402 (A102 silent 12.8%)	ND	ND	ND
prM	742 (G102R 9.3%)	ND	ND	ND
E	1704 (Q256 silent 5.2%) 1707 (E257 silent 5.2%) 1762,1763 (N276G 8.9%) 2170 (M411L 11.8%)	ND	1714 (M260L 32.8%) 1782 (H282Q 39.1%)	1541 (E202G 21.9%) 1714 (M260L 37.2%) 1997 (V354A 5.2%)
NS1	2464 (C15R 12.5%)	3441 (E340D 10.8%)	3234 (G271 silent 34.2%)	3234 (G271 silent 26.0%)
NS2A	**3482 (H2P 50.9%**)	ND	ND	ND
NS2B	4412 (T94I 15.8%) 4472 (I114T 9.5%)	4452 (S107 silent 5.3%)	ND	4412 (T94I 8.4%) 4452 (S107 silent 6.4%) 4472 (I114T 7.3%)
NS3PRO	ND	ND	4530 (V3 silent 34.2%)	4530 (V3 silent 37. 0%)
NS3HEL	5197 (V226M 15.1%) 6061 (E514K 5.4%) 6134 (R538K 5.4%)	6061 (E514K 5.7%)	5418 (Y299 silent 5.5%)	5418 (Y299 silent 9.5%)
NS4A	ND	ND	ND	ND
2K	ND	ND	ND	ND
NS4B	ND	ND	ND	ND
NS5MT	8026 (N153H 10.5%) **8365 (G266R 50.3%**)	ND	7658 (K30R 31.9%) 8366 (G266V 33.9%)	8366 (G266V 27.5%)
NS5POL	ND	ND	10060 (E831Q 6.5%) 10160 (Q864R 25.7%)	8471 (K301R 46.3%) 10060 (E831Q 44.9%) **10160 (Q864R 71.3%**)
3′-UTR	10414 (A to T 99.7%)	10414 (A to T 80.9%)	10414 (A to T 32.7%)	ND

^*a*^ The supernatant of HEL(DENV4)-DENV2 (3) RNA-electroporated BHK-21 cells was used.

^*b*^ The cutoff for inclusion is >5% abundance. The numbers refer to the nucleotide localization (1 to 10723) in the HEL-chimeric DENV2 RNA, followed by the corresponding original amino acid, location, mutated amino acid, and frequency of occurrence shown within parentheses. Bold numbers and letters mean >50% frequency of amino acid alteration. ND, not detected.

Similarly, DENV3 HEL-replacing DENV2 induced various mutations in E (18%), NS3PRO (26%–38%), NS3HEL (5%–15%), and 3’UTR (6%) (Table 4). After infecting C6/36 cells, K112 mutations in NS3PRO increased from 26% to 99.8% frequency, while the other mutations disappeared and new mutations appeared: high-frequency mutations at D223N (99.9%) in HEL as well as multiple lower-frequency mutations in prM (17%–53%), E (7%–53%), NS2B (7%), NS5MT (18%), and 3’UTR (23%) (Table 4). These results suggest that the viral amino acid sequences were selected again to efficiently replicate and/or correctly interact with other viral proteins in C6/36 cells.

**TABLE 4:** Nucleotide (amino acid) alteration in DENV3 HEL-chimeric DENV2^*c*^

Host cell	BHK-21	C6/36	BHK-21	C6/36
RNA	HEL(DENV3)-DENV2	HEL(DENV3)-DENV2 ^*a*^	pHEL(DENV3)-DENV2	pHEL(DENV3)-DENV2^*b*^
Virus collection	30 days p.e.	15 days p.i.	50 days p.e.	15 days p.i.
5′-UTR	ND	ND	ND	ND
C	ND	ND	ND	ND
prM	ND	737 (G100E 20.5%) **836 (F133Y 53.4%**) 870 (I144M 17.3%)	**737 (G100E 98.3%**)	**737 (G100E 99.7%**)
E	1651 (T239S 17.9%)	1142 (T69I 7.9%) 2152 (M406V 6.7%) **2203 (G426R 52.5%**)	1483 (M183V 17.7%) 1725 (A263 silent 5.0%)	ND
NS1	ND	ND	ND	ND
NS2A	ND	ND	3690 (G71 silent 8.0%)	ND
NS2B	ND	4472 (I114T 7.3%)	4333 (S68T 5.2%)	ND
NS3PRO	4796 (G92E 37.5%) 4856 (K112I 25.9%)	**4856 (K112T 99.8%**)	4855 (K112Q 27.9%)	ND
NS3HEL	5633 (D371V 6.0%) 6227 (R569L 9.7%) 6358 (D613Y 15.0%) 6359 (D613V 5.1%)	**5695(D223N 99.9%**)	5085 (N189K 12.3%) 5921 (Q467R 31.2%)	**5085 (N189K 99.5%**) 5481 (P320 silent 22.4%) **5921 (Q467R 99.7%**)
NS4A	ND	6546 (T56 silent 5.1%)	ND	ND
2K	ND	ND	ND	ND
NS4B	ND	ND	ND	7494 (A222 silent 6.7%)
NS5MT	ND	7586 (I5T 17.7%)	ND	ND
NS5POL	ND	ND	ND	ND
3′-UTR	10401 (10398^DV2^) (T to C, 6.0%)^*d*^	10402 (10399 ^DV2^) (A to G 23.4%)	10426 (10423 ^DV2^) (T to C 29.8%)	10426 (10423 ^DV2^) (T to C 99.3%)

^*a*^ The supernatant of HEL(DENV3)-DENV2 RNA-electroporated BHK-21 cells was used.

^*b*^ The supernatant of pHEL(DENV3)-DENV2 RNA-electroporated BHK-21 cells was used.

^*c*^ The cutoff for inclusion is >5% abundance. The numbers refer to the nucleotide localization (1 to 10726) in the chimeric DENV2 RNA, followed by the corresponding original amino acid, location, mutation, and frequency of occurrence shown within parentheses. Bold numbers and letters mean >50% frequency of amino acid alteration. ND, not detected.

^*d*^ ^DV2^ The nucleotide number corresponds to the DENV2 sequence.

### INT-chimeric viruses could efficiently replicate without acquiring mutations but accumulated various mutations at later time points

One of the INT-chimeric RNA electroporations showed more severe cytotoxicity to BHK-21 cells than the other two INT chimeras and did not induce a mutation at 10 days p.e. (Table 5). After the former INT virus was infected to C6/36 cells, only small numbers (6%–8%) of E mutations occurred and no amino acid alterations in NS proteins were observed, indicating that there is no requirement to change the chimera RNA sequence for efficient replication in both BHK-21 and C6/36 cells. In contrast, the latter two INT-chimeric viruses could be cultured for longer times and acquired mutations in a variety of regions, including E, NS2A, NS2B, NS3HEL, NS4B, NS5MT, and 3’UTR (Table 5). Between these two less cytotoxic INT-chimeric viruses, the common mutations were only seen at NS3HEL (R186K). Since the INT-chimeric virus without acquiring mutations could expand efficiently and have strong cytotoxicity, the mutations in the latter two INT-chimeric viruses are not purportedly required for the compensatory mechanism. Those mutations are potentially by-products induced by the INT replacement for maintaining the relationship among viral proteins.

**TABLE 5:** Nucleotide (amino acid) alteration in INT-chimeric DENV2^*b*^

Host cell	BHK-21	BHK-21	BHK-21	C6/36
RNA	INT(DENV4)-DENV2(1)	INT(DENV4)-DENV2(2)	INT(DENV4)-DENV2(3)	INT(DENV4)-DENV2(3)^*a*^
Virus collection	38 d. p.e.	34 d. p.e.	10 days p.e.	11 days p.i.
5′-UTR	ND	ND	ND	ND
C	ND	ND	ND	ND
prM	ND	ND	ND	ND
E	2300 (M455K 17.0%)	**1753 (S373P 97.3%**)	ND	1145 (T70I 7.4%) 1193 (Q86R 5.7%) 1380 (E148A 8.4%)
NS1	ND	ND	ND	ND
NS2A	ND	**3825 (I116G 97.7%**)	ND	ND
NS2B	ND	4199 (K23R 17.7%)	ND	ND
NS3PRO	ND	ND	ND	ND
NS3HEL	5031 (R170S 22.1%) 5060 (D180V 18.2%) 5078 (R186K, 7.2%)	**5078 (R186K 98.3%**) 5730 (D403 silent 9.4%)	ND	5505 (S328 silent 6.6%)
NS4A	ND	ND	ND	ND
2K	ND	ND	ND	ND
NS4B	7468 (T215A 17.3%)	7052 (M76T, 11.4%)	ND	ND
NS5MT	ND	7849 (L94V, 5.5%)	ND	ND
NS5POL	ND	8892 (L440 silent, 6.4%) 9444 (G625 silent, 5.7%)	ND	ND
3′-UTR	10399 (A to G, 6.7%) 10698 (A to C, 7.5%)	ND	ND	ND

^*a*^ The supernatant of INT(DENV4)-DENV2 (3) RNA-electroporated BHK-21 cells was used.

^*b*^ The cutoff for inclusion is >5% abundance. The numbers refer to the nucleotide localization (1 to 10723) in INT-chimeric DENV2 RNA, followed by the corresponding original amino acid, location, mutated amino acid, and frequency of occurrence shown within parentheses. Bold numbers and letters mean >50% frequency of amino acid alteration. ND, not detected.

### DENV3 PRO-replacing DENV1 induced a high-frequency mutation (99.5%) at NS3HEL (P364S)

RNAseq revealed that P364S at HEL was induced as a single high-frequency mutation in DENV3 PRO-replacing DENV1-chimeric viruses (Table 6). Other amino acid mutations occurred at NS2B (15%) and NS4A (17%). Therefore, P364S at HEL mutation was potentially a compensatory mutation to improve the replication efficiency. We created DENV3 PRO-replacing DENV1 cDNA containing the P364S mutation in HEL. The transcribed 5’ cap RNA was electroporated into BHK-21 cells. It was revealed that the reproduced viruses continuously grew without disappearance after initial replication (~6 days) and quickly expanded to all cells at 18 days p.e. (Fig. 7). With the exception of the mutation at E (E204K), all were lower-frequency mutations (6%–18%) in C, NS2B, NS4B, and NS5MT. These did not have any overlap with the mutations in the PRO (DENV3)-DENV1-chimeric viruses, except the NS2B (I114T) mutation (Table 6). After infecting the PRO-chimeric P364S-derived viruses to C6/36 cells, the I114T mutation disappeared (Table 6). These results confirm the sole compensatory role of P364S mutation in HEL in reviving the replication competency of the PRO-replaced chimeric RNA.

**TABLE 6:** Nucleotide (amino acid) alteration in WT DENV1 versus DENV3 PRO-chimeric DENV1^*c*^

Host cell	C6/36	BHK-21	BHK-21	C6/36
RNA	WT DENV1^*a*^	PRO(DENV3)-DENV1	PRO(DENV3)-DENV1-P364S	PRO(DENV3)-DENV1-P364S^*b*^
Virus collection	5 days p.i.	30 days p.e.	50 days p.e.	10 days p.i.
5′-UTR	ND	ND	ND	ND
C	ND	ND	425 (T111A 17.7%)	425 (T111A 34.5%)
prM	ND	ND	ND	ND
E	1118 (E64K 5.7%) 1416 (T163I 13.9%) **1538 (E204K 68.4%**) 1620 (E231G 12.4%) 1686 (V253A 6.7%) 1764 (T279K 7.9%) 2314 (G462 silent 13.9%)	ND	**1538 (E204K 85.5%**)	**1538 (E204K 94.9%**)
NS1	2803 (V128 silent 6.0%)	ND	ND	ND
NS2A	3778 (V101 silent 5.8%)	ND	ND	ND
NS2B	ND	4470 (I114T 15.3%)	4323 (A65V 9.0%) 4379 (M84L 17.0%) 4470 (I114T 8.0%)	4323 (A65V 22.5%) 4379 (M84L 25.5%)
NS3PRO	ND	ND	ND	ND
NS3HEL	6022 (T501 silent 5.3%)	5437 (G306 silent 6.1%) **5609 (P364S 99.5%**)	ND	6034 (I505 silent 5.1%)
NS4A	ND	7270 (V155 silent, 5.1%) 7310 (D169N 17.4%)	ND	ND
2K	ND	ND	ND	ND
NS4B	ND	ND	7065 (D80G 7.2%)	ND
NS5MT	ND	ND	7577 (T2S 5.6%) 7748 (T58A 10.7%)	7748 (T58A 14.4%)
NS5POL	8529 (M319R 7.2%) 10054 (T827 silent 5.3%)	ND	ND	ND
3′-UTR	10540 (A to G 5.2%)	ND	ND	10329 (G to A 6.5%)

^*a*^ The supernatant of WT DENV1 RNA-electroporated BHK-21 cells (6 days p.e.) was used.

^*b*^ The supernatant of PRO(DENV3)-DENV1-P364S RNA-electroporated BHK-21 cells was used.

^*c*^ The cutoff for inclusion is >5% abundance. The numbers refer to the nucleotide localization (1 to 10735) in DENV1 or the chimeric DENV1 RNA, followed by the corresponding original amino acid, location, mutated amino acid, and frequency of occurrence shown within parentheses. Bold numbers and letters mean >50% frequency of amino acid alteration. ND, not detected.

### Partly DENV1 HEL-replacing DENV2 induced a compensatory mutation at NS2B (S68T)

DENV1-pHEL (part of HEL, 170–514 a.a.)-replacing DENV2 induced a single high-frequency amino acid mutation (96%) at S68T in NS2B (Table 7). There was another high-frequency (92%) mutation corresponding to DENV2 10414 nucleotide position at 3’ UTR, similarly seen in DENV4 HEL-replacing DENV2 progenies. There were two other mutations that did not cause amino acid alterations (silent mutation) with lower frequencies (13%–14%) in NS3HEL and NS5MT. In order to test the effect of the NS2B mutation, DENV1 pHEL-replacing chimeric DENV2 cDNA containing S68T mutation in NS2B was created. The transcribed 5’ cap RNA was electroporated into BHK-21 cells. The viral replication was quicker, expanding to all cells at 14 days (Fig. 7), confirming the role of the NS2B mutation in compensating for the replication inefficiency derived from the HEL (DENV1)-replaced DENV2. The RNAseq data showed that no amino acid mutation appeared during replication in BHK-21 cells. The 10414-nucleotide mutation at 3’ UTR was only detected at 6%. After infecting C6/36 cells, the 10414-nucleotide mutation at 3’UTR disappeared and low-frequency mutations newly appeared at NS2B (5%), NS5POL (5%), and 3’UTR (7%).

**TABLE 7:** Nucleotide (amino acid) alteration in DENV1-partly replaced HEL-chimeric DENV2^*b*^

Host cell	BHK-21	BHK-21	C6/36
RNA	pHEL (DENV1)-DENV2	pHEL (DENV1)-DENV2-S68T	pHEL (DENV1)-DENV2-S68T^*a*^
Virus collection	50 days p.e.	22 days p.e.	15 days p.i.
5′-UTR	ND	ND	ND
C	ND	ND	ND
prM	ND	ND	ND
E	ND	ND	ND
NS1	ND	ND	ND
NS2A	ND	ND	ND
NS2B	**4333 (S68T 95.7%**)	ND	4412 (T94I 5.0%)
NS3PRO	ND	ND	ND
NS3HEL	5649 (L376 silent 14.3%)	ND	ND
NS4A	ND	ND	ND
2K	ND	ND	ND
NS4B	ND	ND	ND
NS5MT	8172 (K200 silent 13.3%)	ND	ND
NS5POL	ND	ND	8902 (E444K 5.2%)
3′-UTR	10417(10414^DV2^) (A to T 92.0%)^*c*^	10417(10414^DV2^) (A to T 5.7%)	10405(10402 ^DV2^)(T to C 7.3%)

^*a*^ The supernatant of pHEL (DENV1)-DENV2-S68T RNA-electroporated BHK-21 cells was used.

^*b*^ The cutoff for inclusion is >5% abundance. The numbers refer to the nucleotide localization (1 to 10726) in the chimeric DENV2 RNA, followed by the corresponding original amino acid, location, mutation, and frequency of occurrence shown within parentheses. Bold numbers and letters mean >50% frequency of amino acid alteration. ND, not detected.

^*c*^ ^DV2^ The nucleotide number corresponding to the DENV2 sequence.

**TABLE 9:** Nucleotide (amino acid) alteration in DENV4 pHEL-replacing DENV2 ^*c*^

Host cell	BHK-21	BHK-21
RNA	pHEL (DENV4)(170–573 aa)-DENV2 ^*a*^	pHEL(DENV4)(170–571 aa)-DENV2 ^*b*^
Virus collection	38 days p.e.	34 days p.e.
5′-UTR	ND	ND
C	ND	ND
prM	ND	ND
E	**2104 (N390H 99.9%**)	1185 (K83N, 6.8%) 1964 (E343G 5.4%)
NS1	ND	2556 (A45 silent 23.6%)
NS2A	ND	ND
NS2B	4412 (T94I 8.2%)	4412 (T94I 6.0%)
NS3PRO	ND	ND
NS3HEL	ND	ND
NS4A	ND	ND
2K	ND	ND
NS4B	ND	ND
NS5MT	8308 (H247Y 7.4%)	ND
NS5POL	**10160 (Q864R 70.1%**)	ND
3′-UTR	10414 (A to T 98.9%)	10414 (A to T 77.9%)

^*a*^ pHEL: partly HEL-replaced (NS3 170–573 a.a.).

^*b*^ pHEL: partly HEL-replaced (NS3 170–571 a.a.).

^*c*^ The cutoff for inclusion is >5% abundance. The numbers refer to the nucleotide localization (1 to 10723) in the chimeric DENV2 RNA, followed by the corresponding original amino acid, location, mutation, and frequency of occurrence shown within parentheses. Bold numbers and letters means >50% frequency of amino acid alteration. ND, not detected.

### 3’UTR replacements in DENV2 by the corresponding from DENV3 or DENV4 retained efficient replication and even increased cytotoxicity in the plaque assay, compared with WT DENV2

**Fig 8:**
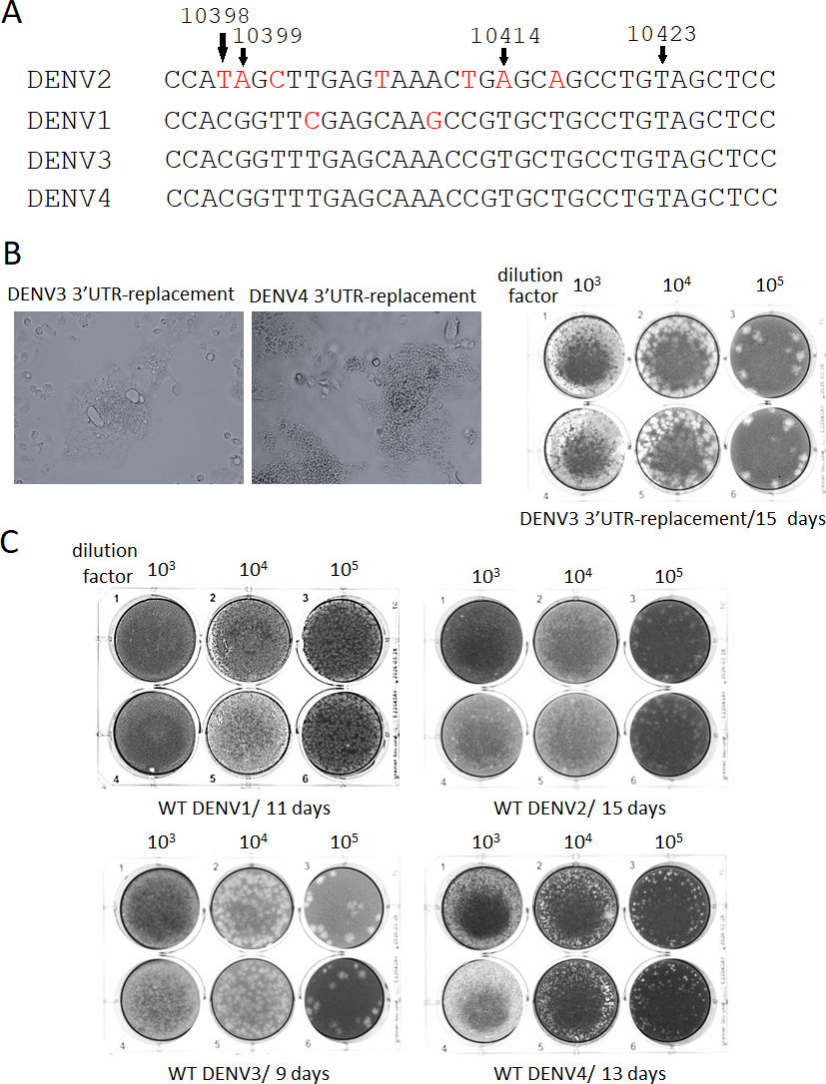
DENV2 3’ UTR was replaced by the corresponding from DENV3 or DENV4. (**A**) 3’UTR (around 10414) nucleotide sequence variations were compared. The red-colored nucleotides are different from the corresponding nucleotides in other serotypes. (**B**) C6/36 cells were infected with supernatants of BHK-21 cells at 10 days post electroporation of 3’UTR-replacing DENV2 RNA (DENV3 3’UTR-DENV2 or DENV4 3’UTR-DENV2). Light microscopic views were taken at 5 days p.i. (left). Plaque assay with LLC/MK2 cells was done with the supernatant of DENV3 3’UTR-replacing DENV2 (right). The plaque sizes were bigger than the WT DENV2 (Fig. 8C). (**C**) Plaque assays were performed using one of WT DENV1–4.

The comparison of 3’UTR nucleotides at 10414 and its surroundings (~35 nucleotides) among serotypes showed that these regional sequences are fully conserved between DENV3 and DENV4, while DENV2 has seven different nucleotides and DENV1 has two differences (Fig. 8A). DENV1 or DENV4 HEL-replacement caused A to T mutation at 10414 (Tables 3 and 7), while DENV3 HEL-replacement induced mutations T to C at 10398, A to G at 10399 or T to C at 10423 (Table 4). All these mutations except 10423 correspond to the 3’ UTR sequences in the HEL-replaced serotypes. Therefore, the interaction between 3’UTR and NS3HEL is considered. In order to examine the role of 3’UTR in interaction with NS3HEL, the whole 3’UTR from DENV3 or DENV4 was replaced in the infectious WT DENV2. These *in vitro* transcribed 5’ cap RNAs efficiently replicated after being electroporated into BHK-21 cells, comparable to WT DENV2 (Fig. 2 and 7). These 3’UTR-chimeric viruses induced syncytial change in C6/36 cells at 5 days and cytotoxicity to LLC/MK2 cell in plaque assay (Fig. 8B). In particular, the DENV3-3’UTR-chimeric virus made larger plaques than WT DENV2 did (Fig. 8B and C), suggesting that the 3’UTR-replacement induced more replication competence. RNAseq analysis showed that no amino acid alteration through NS proteins as well as no nucleotide mutation at 3’UTR occurred in 3’UTR-replacing chimeric viruses from DENV3 or DENV4 during replication in BHK-21 cells (Table 8). After infecting these viruses to C6/36 cells, DENV3-3’UTR chimera only showed low frequencies of amino acid mutations at NS5MT (12%–15%) (Table 8). The data suggests that the replaced 3’UTR is not affecting NS3HEL protein sequences. Among WT DENV1–4, the largest plaque forming ability was shown by WT DENV3 and the smallest was by WT DENV2 (Fig. 8C). It is conceivable that 3’UTR RNA itself affects the replication efficiency and DENV3-3’UTR contributed to more efficient replication, resulting in the larger plaque formation via more cytopathic effects.

**TABLE 8:** Nucleotide (amino acid) alteration in 3’UTR-replaced DENV2^*c*^

Host cell	BHK-21	C6/36	BHK-21	C6/36
RNA	3’UTR(DENV3)-DENV2	3’UTR(DENV3)-DENV2^*a*^	3’UTR(DENV4)-DENV2	3’UTR(DENV4)-DENV2^*b*^
Virus collection	10 days p.e.	10 days p.i.	10 days p.e.	10 days p.i.
5′-UTR	ND	ND	ND	ND
C	ND	ND	177 (Q27H 6.1%) 256 (L54I 10.2%)	256 (L54I 12.1%)
prM	ND	ND	ND	ND
E	1753 (S273P 10.1%)	ND	2013 (T359 silent 5.7%)	1705 (E257Q 10.9%)
NS1	ND	2847 (E142D 9.4%)	3273 (T284 silent 8.4%)	3273 (T284 silent 14.0%)
NS2A	ND	ND	ND	ND
NS2B	ND	ND	4452 (S107 silent 6.8%)	ND
NS3PRO	ND	ND	ND	ND
NS3HEL	ND	6340 (L607 silent 12.9%)	ND	ND
NS4A	ND	6513 (S46 silent 10.7%)	ND	ND
2K	ND	ND	ND	ND
NS4B	ND	7018 (L65 silent 8.9%)	ND	7212 (A129 silent 7.0%)
NS5MT	ND	7670 (Q34L 11.9%) 8241 (A224 silent 5.7%) 8317 (A250T 14.6%)	ND	ND
NS5POL	ND	10080 (P837 silent 10.0%)	ND	ND
3′-UTR	ND	ND	ND	ND

^*a*^ The supernatant of 3’UTR (DENV3)-DENV2 RNA-electroporated BHK-21 cells was used.

^*b*^ The supernatant of 3’UTR (DENV4)-DENV2 RNA-electroporated BHK-21 cells was used.

^*c*^ The cutoff for inclusion is >5% abundance. The numbers refer to the nucleotide localization (1 to 10272) in DENV2 RNA (5’UTR~NS5, except the replaced 3’UTR), followed by the corresponding original amino acid, location, mutation, and frequency of occurrence shown within parentheses. Bold numbers and letters means >50% frequency of amino acid alteration. ND, not detected.

## DISCUSSION

The details remain unknown on how NS3, NS5, and membrane integral NS2A, 2B, 4A, and 4B proteins coordinately work for a series of replication processes. In this study, the NS3FULL replacement did not show replication through all time points, while either PRO- or HEL-replaced chimeras replicated from earlier time points (2–6 days p.e.), albeit with delayed viral expansion or the disappearance of the infected cells after the initial replication period. This is a contrary result to NS5 replacement, in which NS5FULL replacement is less attenuated than either MT or POL domain replacement (23), suggesting that NS3 may be more involved in interactions with other NS proteins, while NS5 may play a more independent role in the replication process. Since NS5 forms dimer (25), the full-length NS5 replacement does not cause a problem for dimerization.

HEL (DENV4)-replaced chimeric DENV2 RNAs could replicate slowly and gradually expand to cells, while PRO (DENV4)-replaced DENV2 caused the disappearance of the chimeric virus progenies after 6 days p.e. However, the other serotypic HEL or PRO replacements showed different results; HEL (DENV1)-replaced DENV2 could not expand the viral progenies and disappeared eventually, whereas PRO (DENV3)-replacing DENV1 expanded after 22 days p.e. It was further found that the partial HEL replacement (pHEL) by DENV1 (N-terminal replacement; 170–514 a.a., excluding C-terminal 515–619 a.a.) could make the progeny expand after 26 days p.e. These sudden viral expansions at later time points may indicate the involvement of compensatory mutations in the recovery of replication competence. The RNAseq data revealed the high-frequency NS2B mutation (S68T), with which the pHEL(DENV1)-chimeric RNA replicated efficiently. The pHEL-chimeric RNA with S68T at NS2B showed faster replication, confirming the role of the NS2B mutation in compensation for the N-terminal HEL replacement.

Viral progenies from HEL (DENV3 or DENV4)-replaced infectious RNA could be slowly reproduced, accompanied by diversely localized and various frequency mutations in NS proteins, in addition to prM and E proteins. A possible explanation for the variously located mutations with diversified frequencies is a sequential occurrence of the mutations, induced by protein–protein interactions. The first mutation leads to a second one, which further causes a third one, and so on, in order to correct the protein–protein interactions. In this regard, all observed mutations are not necessary to regain the impaired functions by compensatory mutations. It is considered that the accumulated mutations are either (1) necessary as a compensatory function to recover the viral life cycle or (2) induced by protein–protein interactions or mechanism(s) other than those required for replication fitness.

**Fig 9:**
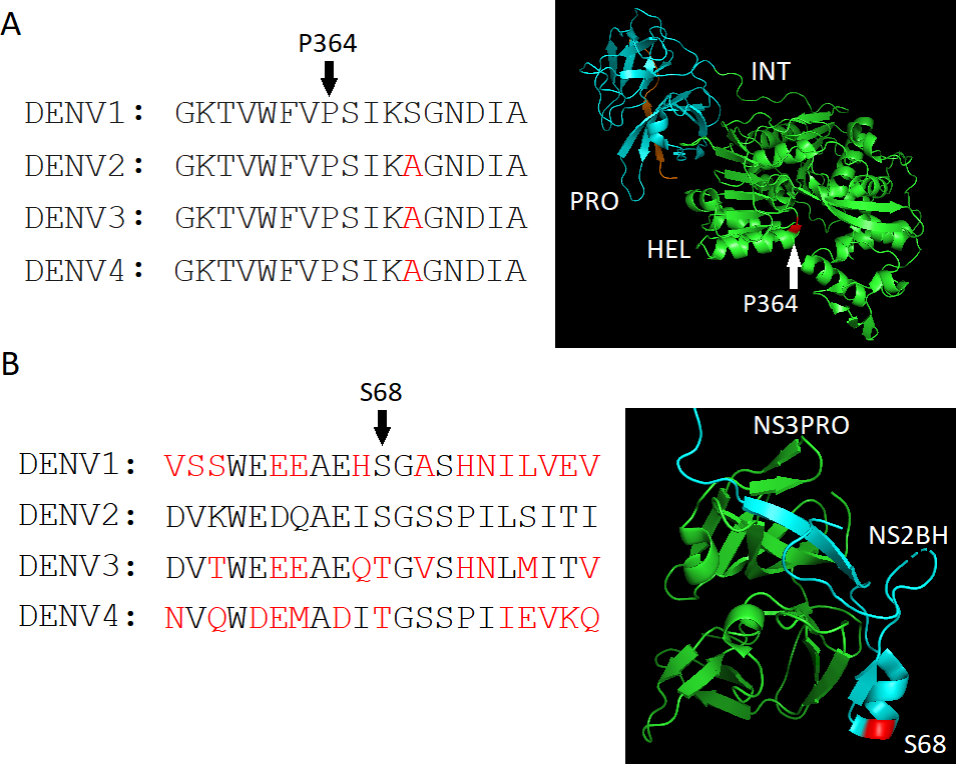
Localizations of the mutated amino acids that compensate the attenuation by PRO or HEL replacement-induced attenuation. (**A**) P364 amino acid and the surrounding region in HEL are compared among DENV serotypes (left). Amino acid variations are minimal in this region. The red-colored letters are the different amino acids compared with the DENV1 sequence. P364 (the red point) localizes at the dip from the surface area (right) (PDB file: 2VBC). (**B**) S68 amino acid and the surrounding region in NS2B are compared among DENV serotypes (left). The red-colored letters show the different amino acids compared with DENV2. The S68 in NS2B (the red point) localizes outside for binding to PRO (right) (PDB file: 4M9K).

The PRO (DENV3)-replaced viral progeny in DENV1 RNA could expand at a later time point by acquiring the compensatory mutation at P346S in HEL. The relationship between PRO and HEL was also observed in the HEL replacement. NS3PRO mutations at K112T (99.8%) and K112Q (28%) were seen in DENV3 HEL-replacing DENV2 (Table 4). P364S in HEL is localized at the dip area from the protein surface area (Fig. 9A), suggesting that the mutation may not directly affect the binding to the counterpart, but may change the HEL domain structure. S68T at NS2B is positioned at the surface of the hydrophilic region but distant from PRO-binding region (Fig. 9B), suggesting no correlation with PRO function, although since NS2B joins the replication compartment with the ER membrane, the NS2B hydrophilic region’s mutation may affect communication between NS3HEL and the replication compartment formed by NS2B.

NS4B was reported as having a critical interaction with NS3 for DENV replication (17). Another report described that NS4B enhanced HEL function for dissociating NS3 from RNA (26, 27). However, the detailed sequence requirement for NS3-NS4B interaction remains unclarified. In this study, none of the HEL replacements induced NS4B amino acid alteration (Tables 3, 4, 7, and 9), although NS3INT replacement caused NS3HEL (7%–98%) and NS4B (11%–17%) mutations (Table 5). It is possible that the altered NS3INT sequence affects HEL and NS4B interaction. Further analysis, such as mutagenesis-based positional analysis, is necessary for clarifying the interaction between NS3 and NS4B.

INT-replaced chimeric RNA could replicate more efficiently than other chimeric RNAs without inducing a mutation. However, mutations were accumulated through the extended culturing INT-chimeric viruses. The results raise a question of whether various mutations could simply be accumulated by culturing for a longer time. Since WT DENV2 is difficult to continue culturing in BHK-21 cells due to strong cytotoxicity, the sequence data at later time points is not available. However, WT DENV1 collected at an earlier time point (6 days after the electroporation into BHK-21 cells plus 5 days infection to C6/36 cells) showed accumulations of various amino acid alterations in E protein, including a high-frequency one at 68% (Table 6), suggesting that regardless of time points, high-frequency mutations can be quickly accumulated in viral populations. Conversely, the long-time cultured (50 days p.e.) chimeric viruses (DENV1 pHEL-replacing DENV2) showed few amino acid alterations (Table 7), also suggesting that longer culturing viruses do not simply accumulate broad mutations. Therefore, the mutations occurring in INT-replaced progenies could be induced by interactions between viral proteins for reasons other than a compensatory requirement.

Replicon data showed that NS3-chimeric replacements except INT chimera are barely able to replicate (~7 days) (Fig. 5B). However, the infectious HEL or PRO replacement could replicate at 2 days p.e. HEL-replaced infectious RNA gradually expanded without a compensatory mutation, while the PRO-replaced chimeras disappeared after 6 days p.e. and a compensatory mutation was required for regaining replication expansion at a later time point. Therefore, the replicon data is not simply applicable to the mechanism of the HEL- or PRO-replaced infectious RNA. It was reported that the major difference in replication characteristics of infectious viruses compared with replicons was the distribution of the viral double-stranded RNA, which was correlated to the rearrangement of the ER and Golgi apparatus (28, 29). It was suggested that the replicon could induce less rearrangement of the ER and Golgi apparatus, compared with infectious RNA, and therefore, it was suggested that the replicon data only reflects the latent period of the initial replication process prior to the later stage of excessive (+) sense RNA synthesis (28). It is speculated that NS3FULL, PRO, and HEL replacement were all less efficient in the initial replication (latent period) than WT or NS3INT chimera. Since PRO (DENV4)- or HEL (DENV4)-chimeric DENV2 RNAs can be characterized differently based on the viral expansion or disappearance course after the initial replication, the difference between these chimeras must rather exist in rearranging the ER–Golgi apparatus to form the replication compartment. It is considered that the more severe replication incompetence (the viral disappearance) was due to incomplete building of the replication compartment with rearranged ER and Golgi apparatus and that NS2B-HEL and NS3PRO-HEL interactions are involved in it. The compensatory mutation was required for recovering this ER–Golgi rearrangement in PRO (DENV3)-replaced DENV1 as well as pHEL (DENV1)-replaced DENV2, while the ER–Golgi rearrangement could be slowly induced without compensatory mutation in HEL (DENV4 or DENV3)-replaced chimeric RNAs. Reproduced viral progenies from PRO- or HEL-replaced chimera still did not cause cytotoxicity, while the INT-replaced one induced syncytial change of C6/36 cells as well as plaque forming cytotoxicity in LLC/MK2 cells, suggesting the importance of the initial latent period in viral replication efficiency.

The varied frequency A to T nucleotide mutations at 10414 position in 3’UTR were induced in HEL (DENV1 or DENV4)-replaced chimeric DENV2. 10398 and 10399 mutations in 3’UTR were also induced in HEL (DENV3)-replaced DENV2. These 3’UTR mutations may not be important as a compensated role for the HEL replacement since they were not so high-frequency in many recovered progeny viruses and even disappeared after infecting C6/36 cells. The addition of NS2B S68T mutation in the pHEL (DENV1) chimera RNA showed a faster replication without a significant induction of 10414 in 3’ UTR (6%, Table 7). It is possible that these mutations in 3’UTR do not correlate to NS3HEL but increase replication competence under another mechanism. The replacement of 3’UTR with corresponding DENV3 or DENV4 did not induce an amino acid mutation at NS proteins nor any nucleotide mutation at 3’UTR, but induced syncytial morphological change in C6/36 cells as well as increased cytotoxicity in the plaque assay, reflecting increased replication efficiency.

## MATERIALS AND METHODS

### Construction of full-length DENV cDNA, in which NS3FULL, PRO, HEL, or INT domain was replaced

The yeast/*E. coli* shuttle vector plasmid pRS424 was used for cloning the full-length DENV1–4 cDNAs (PRS424-FLDV1–4, gifts from Dr. Falgout, FDA). The replacement (NS3FULL, PRO, HEL, or INT) regions were amplified by PCR, consisting of one to three fragments, including 5’ and 3’ adjacent homologous sequences (>30 nucleotides, primer details are described in the Table S1). The amplified PCR products were mixed with the linearized pRS424-FLDV2 by XhoI (at 5427–5430 nt position) or pRS424-FLDV1 by NheI (at 5877–5890 nt position) to form *Saccharomyces cerevisiae* YPH857 competent cells. The homologous recombination-occurred plasmid-contained *S. cerevisiae* was selected and amplified in Tryptophan-lacked yeast media, followed by transforming and expanding *E. coli* stbl2 competent cells in ampicillin-contained LB media (30, 31). The designed homologous recombination as well as the incomplete replacements happened by chance in yeast cells. These cDNAs sequences were verified by Sanger (Genewiz) and Nanopore sequencings (Plasmidsaurus).

### *In vitro* RNA transcription and electroporation into BHK-21 mammalian cells

Infectious NS3-chimeric DENV cDNAs were linearized by SacII (for DENV1 backbone), or BcgI or SacI (for DENV2 backbone) at the 3’ ends. The linearized cDNAs were used as templates for *in vitro* transcription reaction to synthesize 5’ cap infectious RNAs with SP6 RNA polymerase and m^7^GpppG cap analog (30, 31). Each synthesized RNA (~3 µg) was electroporated into ~1 x 10^6^ BHK-21 cells (American Type Culture Collection), using Amaxa Nucleofector II system (Amaxa). The pulsed cells were immediately spread on a T-25 flask, followed by splitting into T-75 flask at 2 days p.e. Afterward, these cells were continuously cultured by repeated splitting every 4 days using one-third of the trypsinized cells and fresh medium (23).

### IF staining against NS1 for detecting WT or chimeric DENV

The productions of the chimeric viruses were periodically monitored by IF staining against viral NS1 (IFA) using 7E11, a monoclonal anti-DENV NS1 antibody (1:200 dilution, gift from Dr. Falgout, FDA). FITC-labeled goat anti-mouse IgG conjugate was used as a secondary antibody (1:100 dilution, Seracare) to visualize viral NS1 under epifluorescent microscope (Olympus IX-71). Nuclear staining was performed with DAPI (1:400 dilution, KPL) and was compared with cytoplasmic localization of NS1. The rate of viral NS1-positive cells versus total cell numbers in more than six microscopic fields was counted under NIH image software and compared among samples of different NS3 chimeras and testing dates.

### Measurement of viral copy numbers (RT-qPCR) and cytopathic viral titer (plaque assay)

WT or chimeric DENVs in the supernatants of cell cultures were analyzed for their viral copy numbers. Viral RNAs were extracted, using Quick-RNA^TM^-Viral kit (Zymo Research). RT reaction was performed to make the viral cDNAs by ProtoScript II reverse transcriptase (NEB) with random primer and dNTPs (NEB) at 42°C for 2 hours. The copy numbers of cDNAs were measured by qPCR with primer pair amplifying capsid or NS1 region (Table S1) and iTaq Universal SYBR green Supermix (Bio-Rad) in Mic qPCR cycler (Bio Molecular System). The average threshold cycle (Ct) values were converted to viral copy numbers by comparing them with the standard amounts of DENV cDNAs.

Plaque assay was performed in paired wells of six well plates, using LLC/MK2 cells, which were infected for 2 hours by WT or chimeric DENV from serially diluted supernatants in the infected C6/36 cell cultures. After removal from medium, cells were overlaid with DMEM containing 0.9% SeaPlaque agarose (Lonza) and were continuously incubated for 9–15 days at 37°C. Plaques were fixed with 37% formaldehyde (VWR) and were visualized by crystal violet staining.

### Rluc reporter replicon assay

pRS424-Rluc-WT DENV2 plasmid contains Rluc cDNA that is flanked by DENV2 5’ terminal 171 nt at 5’ side and EMCV IRES sequence at 3’ side, followed by C-terminal E (73 amino acids) and the continuing downstream full genome; NS1 ~NS5 and 3’UTR (Fig. 5A). NS3-chimeric Rluc replicons were made by yeast recombination, using PCR-amplified NS3 fragments and XhoI-linearized pRS424-Rluc-DENV2 (22). After SacI-cutting the constructed cDNA plasmids at the 3’ termini, each replicon RNA was *in vitro* transcribed with a cap analog under Sp6 polymerase reaction and was electroporated into BHK-21 cells. Cells were lysed at 4, 24, 48, 72, 96, 120, 144, and 168 hours p.e. with 250 µL of 1 x Rluc lysis buffer (Promega). Rluc activities were measured in duplicated lysed samples (50 µL) using a Centro LB960 luminometer (Berthold Technologies) by injecting 50 µL of 1 x Rluc substrate (Promega) with settings of 10 secs reading and 2 secs delay after the substrate injection.

### RNA extraction from viral particles and RNAseq analysis

Supernatants (~10 mL) were collected from BHK-21 or C6/36 cell cultures in T-75 flasks. Viral particles were precipitated by centrifugation at 15,000 g for 45 min in 40% PEG8000 solution containing 10 mM Tris (pH 8.0), 120 mM NaCl, and 1 mM EDTA (23). After removing the supernatants, the remaining 0.5 mL solutions at the bottom of the tubes, containing the precipitates, were repeatedly mixed with 1.5 mL Trizol-LS (Ambion) and 0.4 mL chloroform. After the centrifugation of these mixed solutions at 12,000 g for 15 mins, the upper layer separated from Trizol/chloroform (bottom layer) was used for ethanol precipitation to collect viral RNA. The ethanol-precipitated pellet was dissolved by Tris (10 mM, pH 8.0)-buffered DEPC water and was purified with RNA Clean & Concentrator^TM^ kit (Zymo Research). Library preparation and RNAseq sequencing were performed at Zymo Research Inc. (Irvine, CA), where each RNA sample (260 ng) was purified using Zymo-Seq RiboFree Total RNA Library kit (Zymo Research). Ribosomal RNA was removed from RNA samples, which were then reverse-transcribed to cDNAs. The produced cDNAs were ligated with P7 adaptor sequence at 3’ end, followed by second strand synthesis and P5 adaptor ligation to the opposite sites of the double-stranded DNAs. After purification by DNA size (300–600 bp) with beads in the kit, index PCR was performed; initial denaturation at 95°C for 10 mins; 16 cycles of denaturation at 95°C for 30 secs, annealing at 60°C for 30 secs, and extension at 72°C for 60 secs; and final extension at 72°C for 7 mins. Successful library construction was confirmed with Agilent’s D1000 ScreenTape Assay on TapeStation. Libraries were sequenced on an Illumina Novaseq to a sequence depth of >30 million read pairs (150 bp paired-end sequencing).

Raw Fastq files created from RNAseq reactions were used for alignment analysis of DENV sequences using Geneious Prime software (Biomatters). Paired-end sequences were made from R1 and *R2* Fastq files. After trimming the 3’ end by BBDuk plugin, the paired-end sequences were aligned to the reference sequence. The created contigs were analyzed for SNPs. Nucleotide alterations at more than 5% frequency were shown.

### Statistical analysis

Measurements of viral copy numbers, viral titers, and Rluc replicon assays were performed with duplicated samples and the average numbers and SD were calculated. Viral NS1-posite cell rates were calculated with six microscopic fields, and the average numbers and SD was calculated. Variation analyzed as significant difference was assessed with one-way ANOVA with Bonferroni post-test, using Prism (Graphpad software).

## Data Availability

The RNAseq data as raw reads are available as fastq files in the NCBI Short Read Archive (SRA) Data/Download Web page (BioProject accession number, PRJNA916891).

## SUPPLEMENTAL MATERIAL

Fig. S1 and S2 and Table S1 jvi.00854-23-s0001.docx NS3 Amino acid alignment figures and primer sequence table.

ASM does not own the copyrights to Supplemental Material that may be linked to, or accessed through, an article. The authors have granted ASM a non-exclusive, world-wide license to publish the Supplemental Material files. Please contact the corresponding author directly for reuse.
