# Supplementary material for: Dengue virus serotypic replacement of NS3 protease or helicase domain causes chimeric viral attenuation but can be recovered by a compensated mutation at helicase domain or NS2B, respectively
Source: J Virol. 2023 Aug 9;97(8):e00854-23. doi: 10.1128/jvi.00854-23 (PMC10506484; doi:10.1128/jvi.00854-23)
Supplement: Fig. S1 and S2 and Table S1 — NS3 Amino acid alignment figures and primer sequence table. [file jvi.00854-23-s0001.docx]

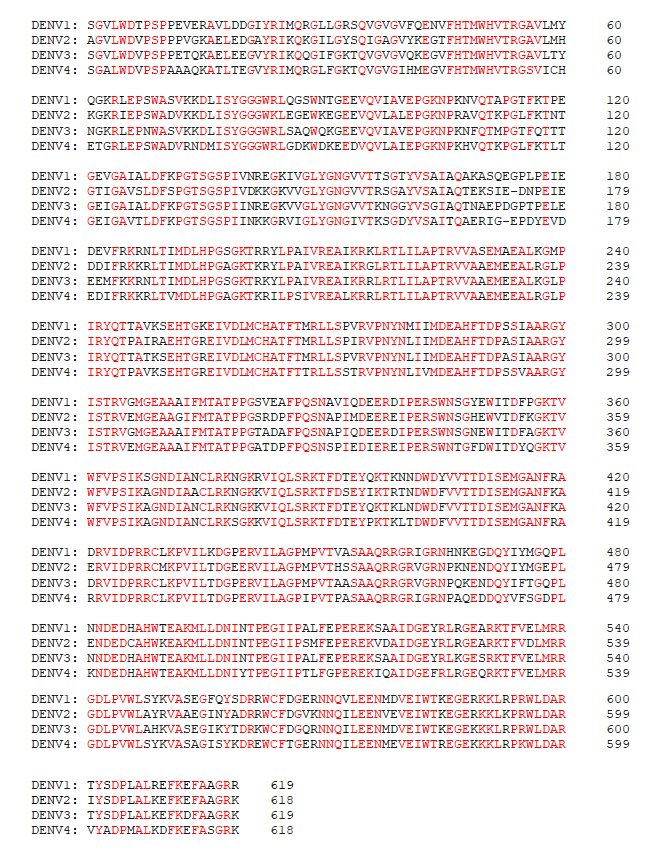


**Supplemental Fig. 1.** Conserved amino acid sequences among DENV four serotypes. The red colored letters are the identical amino acids among the DENV four serotypes. The rate of amino acid identities among all four serotypes is 68 %.


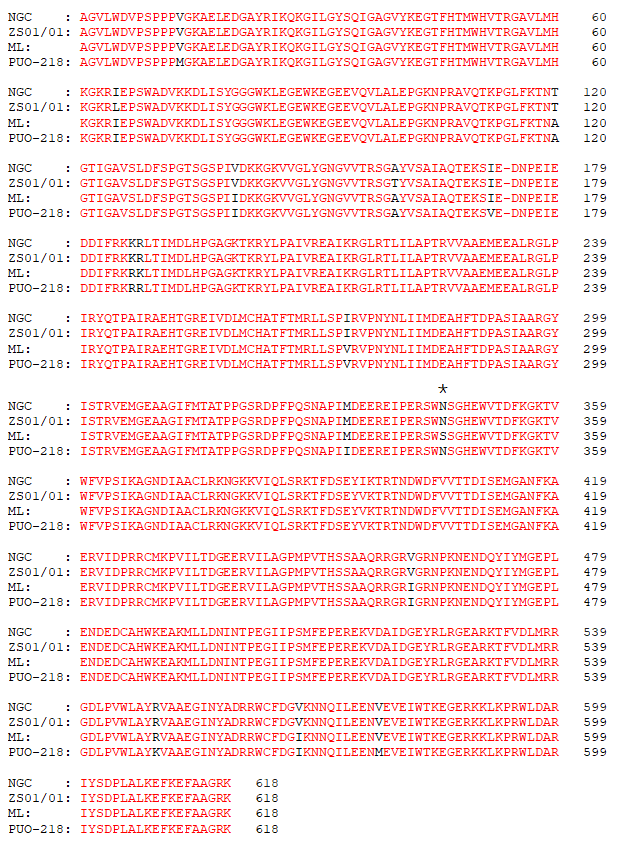


**Supplemental Fig. 2.** Conserved amino acid sequences among four DENV2 strains. The red colored letters are the identical amino acids among the four strains. All of the amino acid variations (black letters) are seen in the four serotypic amino acid differences (supplemental Fig. 1) except for the one labeled with the asterisk (*). The rate of amino acid identities among the four strains is 98 %. NGC strain was used in the experiment.

**Supplemental Table 1.** PCR Primers used for the replacement of NS3FULL, PRO, HEL, or INT as well as qPCR.

| PCR fragment | forward | reverse |
| --- | --- | --- |
| FULL(DENV4)-DENV2 | GCAGCATGGTACCTGTGGGAAGTGAAGAAA  CAACGGTCAGGAGCCCTGTGGGACGTC | AAGCCTACCCATTTCTGTGATTAGGTTCAG  GGTCAGGGACTTCCTTCCACTGGCAAACT |
| PRO(DENV4)-DENV2-1 | GCAGCATGGTACCTGTGGGAAGTGAAGAAA  CAACGGTCAGGAGCCCTGTGGGACGTC | GTCATCTTCGATCTCTGGATTGTCTTCAAT  ACTTTTTTCGGCTTGGGTTATGGCACTG |
| PRO(DENV4)-DENV2-2 | AAAAGTATTGAAGACAATCC (5029F) | GGTTCTAGTCTTGACATACTC (5700R) |
| HEL(DENV4)-DENV2 | AGGAGTGGAGCATATGTGAGTGCTATAGCC  CAGACTGAAAGAATTGGAGAGCCAGAT | AAGCCTACCCATTTCTGTGATTAGGTTCAG  GGTCAGGGACTTCCTTCCACTGGCAAACT |
| INT-(DENV4)-DENV2-1 | CATGTCAGCGGCCATAAAAGA (2970F) | ATCCACTTCATAATCTGGCTCTCCAATTCT  TTCGGCCTGGGCTATAGCACTCACAT |
| INT(DENV4)-DENV2-2 | GCCGAAAGAATTGGAGAGCCAGATTATGAA  GTGGATGATGACATTTTTCGAAAGAG | GGTTCTAGTCTTGACATACTC (5700R) |
| PRO(DENV1)-DENV2-1 | CATGTCAGCGGCCATAAAAGA (2970F) | CCGTTGTTTCTTCACTTCCC (4521R) |
| PRO(DENV1)-DENV2-2 | GGGAAGTGAAGAAACAACGGTCAGGAGTGC  TATGGGACAC | GGATTGTCTTCAATACTTTTTTTAGCTTGA  GCTATGGCAC |
| PRO(DENV1)-DENV2-3 | AAAAGTATTGAAGACAATCC (5029F) | GGTTCTAGTCTTGACATACTC (5700R) |
| PRO(DENV3)-DENV2-1 | CATGTCAGCGGCCATAAAAGA (2970F) | CCGTTGTTTCTTCACTTCCC (4521R) |
| PRO(DENV3)-DENV2-1 | GGGAAGTGAAGAAACAACGGTCTGGCG  TTCTATGGGACGT | GGATTGTCTTCAATACTTTTATTTGTTTGC  GCTATTCCAC |
| PRO(DENV3)-DENV2-2 | AAAAGTATTGAAGACAATCC (5029F) | GGTTCTAGTCTTGACATACTC (5700R) |
| HEL(DENV1)-DENV2 | AGGAGTGGAGCATATGTGAGTGCTATAGCC  CAGACTGAAGCATCACAAGAAGGGCCTCT | AAGCCTACCCATTTCTGTGATTAGGTTCAG  GGTCAGGGATCTTCTTCCTGCTGCGAACT |
| HEL(DENV3)-DENV2 | AGGAGTGGAGCATATGTGAGTGCTATAGCC  CAGACTGAAGCAGAACCAGACGGACCGAC | AAGCCTACCCATTTCTGTGATTAGGTTCAG  GGTCAGGGACTTTCTGCCAGCTGCAAAAT |
| NS2B-NS3FULL (DENV4)-DENV2 | CTAACAACCCTTTCAAGAACCAACAAGAAA AGGTCTTGGCCTCTTAACGAGGGC | AAGCCTACCCATTTCTGTGATTAGGTTCAG  GGTCAGGGACTTCCTTCCACTGGCAAACT |
| NS2B-NS3PRO (DENV4)-DENV2 | CTAACAACCCTTTCAAGAACCAACAAGAAA AGGTCTTGGCCTCTTAACGAGGGC | GTCATCTTCGATCTCTGGATTGTCTTCAAT  ACTTTTTTCGGCTTGGGTTATGGCACTG |
| PRO(DENV3)-DENV1-1 | GGAATCTTGGCCCAAGGAAAG (DENV1-2700F) | TGTTTCTGGGGGGCTGGGTACGTCCCATAG AACGCCAGATCTCTGTTTCTTTTTCTGCC |
| PRO(DENV3)-DENV1-2 | TCTTTGTGTGGTATTTTTGGCAGAAAAAGA  AACAGAGATCTGGCGTTCTATGGGACGTA | CTCGTCCTCAATCTCTGGTAGAGGCCCTTC  TTGTGATGCATTTGTTTGCGCTATTCCAC |
| P364S-PRO-1 | GTGGGTATGGGTGAAGCAGC (DV1-5430F) | GTCATTTCCTGATTTGATGCTTGAAACAAA  CCAGACTGTTTTACC |
| P364S-PRO-2 | GGTAAAACAGTCTGGTTTGTTTCAAGCATC  AAATCAGGAAATGAC | CGCCTTCCTTATTTTGGTTCC (DV1-5900R) |
| S68T-HEL-1 | TGTCCGTTTCCCCACTGTTCT (4000F) | TGACAGGATTGGACTGCTTCCTGTTATCTC  TGCCTGATCTTCCCA |
| S68T-HEL-2 | TGGGAAGATCAGGCAGAGATAACAGGAAGC  AGTCCAATCCTGTCA | GAGTCCTGGCCCTATGATGGC (7200R) |
| 3’UTR(DENV3)-DENV2-1 | CAAACTGGATCAGCATCATCC (8506F) | GTGGCCTGACAGTGTGCCTCTTTTTTACTTCCT  CCGTCTACCACAGGACTCCTGCCTC |
| 3’UTR(DENV3)-DENV2-2 | AGAAGAGAAGAGGAAGAGGCAGGAGTCCTG  TGGTAGACGGAGGAAGTAAAAAAGAGG | CTCACTAAAGGGGCACCCGGGTCGAAGCTG  GAGCTCAGAACCTGTTGATTCAACAGC |
| 3’UTR(DENV4)-DENV2-1 | CAAACTGGATCAGCATCATCC (8506F) | CTGACTTCAATAGCCTTTGGTGTTTGTTGTTGG  TGACTACCACAGGACTCCTGCCTC |
| 3’UTR(DENV4)-DENV2-1 | AGAAGAGAAGAGGAAGAGGCAGGAGTCCTG  TGGTAGTCACCAACAACAAACACCAAAG | CTCACTAAAGGGGCACCCGGGTCGAAGCTG  GAGCTCAGAACCTGTTGGATCAACAAC |
| Capsid-for-PCR | CTGTACAACAGCTGACAAAG (170F) | CCTTCCAATCTCTTTCCTGAA (366R) |
| NS1-for-qPCR | CATGTCAGCGGCCATAAAAGA (2970F) | CACCACTGTGGTTCCTTCGCA (3260R) |

Numbers in the parenthesis mean the viral genome nucleotide position. F or R means forward or reverse, respectively.
